# Comparison of Viable and Heat‐Inactivated *Lacticaseibacillus rhamnosus* IDCC 3201: Anti‐Pathogenic, Anti‐Inflammatory, and Microbiota Modulating Effect

**DOI:** 10.1002/fsn3.71780

**Published:** 2026-04-15

**Authors:** Hayoung Kim, Hanbin Lee, Won Yeong Bang, Haeseong Park, Jin Seok Moon

**Affiliations:** ^1^ Ildong Bioscience Pyeongtaek‐si Republic of Korea

**Keywords:** anti‐pathogenic, ex vivo fermentation, heat‐inactivated, *Lacticaseibacillus rhamnosus*, metabolite profiling, microbiota

## Abstract

*Lacticaseibacillus rhamnosus* is widely studied for strain‐specific antimicrobial and immunomodulatory properties, but comparative data for viable and heat‐inactivated preparations remain limited. This study compared the anti‐pathogenic activity of live *L. rhamnosus* IDCC 3201 and its commercially prepared heat‐inactivated formulation, RHT 3201, and examined their associated microbiota and metabolite profiles in a single‐donor ex vivo fecal fermentation model. Transcript‐level immunomodulatory activity was evaluated separately for RHT 3201 in LPS‐stimulated RAW 264.7 cells. Both preparations inhibited key intestinal pathogens, including *Escherichia coli* , *Staphylococcus aureus* , *Enterococcus faecalis* , and *Salmonella* Typhimurium, although live IDCC 3201 showed stronger inhibition against *E. faecalis* and *S. Typhimurium* . In LPS‐stimulated RAW 264.7 cells, RHT 3201 reduced IL‐1β, IL‐6, COX‐2, and TNF‐α mRNA expression by approximately 39%, 23%, 17%, and 16%, respectively, at 10 × 10^7^ CFU/mL. At the species level in the ex vivo fermentation model, RHT 3201 was associated with a higher relative abundance of *Bifidobacterium pseudocatenulatum* and *Lactobacillus ruminis* , whereas *L. rhamnosus* was detected only in the IDCC 3201 group during the 24‐h culture period. Metabolite profiling also showed distinct product‐associated signatures between the two preparations, with higher lactic acid detected in RHT 3201. Overall, the two preparations showed comparable activity against some pathogens but distinct microbiota‐ and metabolite‐associated profiles in this single‐donor ex vivo system, while anti‐inflammatory activity was assessed only for RHT 3201 at the mRNA level. Additional multi‐donor, protein‐level, and in vivo studies are required to confirm the reproducibility and physiological relevance of these findings.

## Graphical abstract

This graphical abstract illustrates the comparative effects of viable *Lacticaseibacillus rhamnosus* IDCC 3201 and its heat‐inactivated form, RHT 3201. While viable IDCC 3201 showed broad‐spectrum pathogen inhibition and ecological persistence, heat‐inactivated RHT 3201 retained anti‐inflammatory activity and promoted beneficial microbiota, indicating complementary gut‐health benefits through distinct mechanisms.

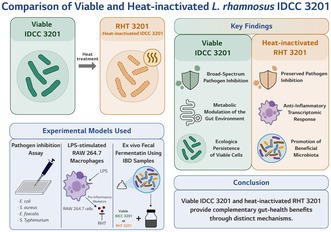

## 1 Introduction

Probiotics are defined as live microorganisms that, when administered in adequate amounts, confer health benefits (Hill et al. 2014). Reported effects of probiotics include modulation of microbial ecology, immune signaling, and resistance to enteric pathogens, although these responses are strain‐ and context‐dependent (Bron et al. 2017; Sanders et al. 2019). Heat‐treated or heat‐inactivated preparations retain nonviable microbial structures and metabolites that may also influence host or microbial systems, while offering improved stability during processing and storage (Piqué et al. 2019).

Certain probiotic strains have been reported to modulate cytokine secretion, support intestinal barrier function, and inhibit pathogenic bacteria (Hardy et al. 2013). Related effects have also been described for heat‐inactivated preparations, but the magnitude and mechanism of these responses vary by strain, manufacturing process, and assay system (Piqué et al. 2019).

Inflammatory bowel disease (IBD), including Crohn's disease and ulcerative colitis, is a general term for a group of conditions characterized by chronic inflammation of gastrointestinal tissues and intestinal barrier dysfunction (Ramos and Papadakis 2019). IBD pathogenesis involves gut dysbiosis, immune dysregulation, and genetic factors, significantly diminishing quality of life (Antunes et al. 2021). Recent evidence has suggested that probiotics, prebiotics, and synbiotics may restore gut microbial balance, alleviate inflammation, and improve intestinal barrier function (Zhang et al. 2021). Fermentation‐based studies using fecal samples from patients with IBD can provide valuable insights into how probiotics influence the gut microbial composition and metabolic environment (Pham and Mohajeri 2018).

Although many probiotic effects are strain‐specific, *Lacticaseibacillus rhamnosus* has been widely investigated for antimicrobial and immunomodulatory properties. Both viable and heat‐inactivated preparations have shown bioactivity in selected experimental systems, but direct interpretation across studies is complicated by strain specificity and differences in processing conditions (Jeong et al. 2020; Kwon et al. 2024; Jalali et al. 2024). Comparative data that evaluate viable and commercially processed heat‐inactivated preparations within the same study remain limited. Therefore, the present study compared live *L. rhamnosus* IDCC 3201 and its heat‐inactivated formulation, RHT 3201, with respect to anti‐pathogenic activity and donor‐derived ex vivo microbiota/metabolite profiles while evaluating transcript‐level immunomodulatory activity separately for RHT 3201 in RAW 264.7 cells.

## 2 Materials and Methods

### 2.1 Bacterial Strains and Culture Conditions

#### 2.1.1 Strain Information and Laboratory Culture Conditions

The probiotic strain *L. rhamnosus* IDCC 3201 (ATCC BAA‐2836), isolated from the feces of breast‐fed infants, was anaerobically cultured in De Man, Rogosa, and Sharpe (BD Difco, Franklin Lakes, NJ, USA) medium at 37°C for 18 h. *L. rhamnosus* IDCC 3201 was heat‐inactivated in an autoclave at 75°C for 2 h.

#### 2.1.2 Large‐Scale Fermentation and Downstream Processing

For the in vitro colonic fermentation experiments, both live *L. rhamnosus* IDCC 3201 and heat‐inactivated *L. rhamnosus* IDCC 3201 (RHT 3201) were prepared. Seed cultures were initiated by inoculating a sterile medium with 1% (v/v) glycerol stock of *L. rhamnosus* IDCC 3201, which was then grown at 37°C in a 2‐L fermenter (pre‐culture). This pre‐culture was used to inoculate an intermediate culture at 10% (v/v) in a 50‐L fermenter under the same conditions. The main fermentation began by introducing 5% (v/v) into a 500‐L fermenter at 37°C and continued until the pH reached 4.8 or lower, yielding sufficient biomass.

After cultivation, the broth was pasteurized at 121°C for 5 min, cooled to 30°C, and the biomass was harvested by centrifugation. The collected cells were subjected to heat treatment at ≥ 70°C for at least 2 h to generate heat‐inactivated biomass. The supernatant was separated and concentrated using a 1‐kL concentrator (≤ 70°C, ≤ 700 mmHg, within 8 h, final volume ≤ 80 L). The heat‐inactivated biomass was blended with 26 kg of excipients and freeze‐dried under controlled conditions (freezing below −30°C and drying at 25°C at 50 mTorr). The dried material was milled at 4000 rpm (screen size: 0.5 mm), sieved through a 60‐mesh sieve, and homogenized to obtain a uniform powder.

To produce live *L. rhamnosus* IDCC 3201 preparations, the same steps were followed, omitting the heat treatment and blending phases.

### 2.2 Study Subject and Fecal Sample Collection

This study was conducted using fecal samples collected from a single adult donor diagnosed with IBD. Repeated in vitro colonic fermentation experiments were performed using this donor‐derived fecal sample and should therefore be interpreted as experimental replicates rather than independent biological replicates. The sample was collected using a dedicated collection kit (AccuStool Collection Kit, AccuGene, Incheon, Korea) and immediately stored at −80°C. The collection of fecal samples was approved by the Institutional Review Board of Seoul Songdo Hospital (approval number: 2021‐004).

### 2.3 Anti‐Pathogenic Activities

Anti‐pathogenic activities were evaluated as previously described by Chae et al. (2022). The anti‐pathogenic activity of *L. rhamnosus* IDCC 3201 and heat‐inactivated RHT 3201 was evaluated against five clinically relevant pathogenic bacteria. Table 1 summarizes the culture conditions for the pathogenic bacteria used in this study. The cell densities of the pathogens were adjusted to final concentrations of 1.5 × 10^6^–10^8^ CFU/mL using the McFarland turbidity standard (BioMérieux, Marcy‐l'Étoile, France) and dispensed into 96‐well microtiter plates. Subsequently, 100 μL of the supernatant from *L. rhamnosus* IDCC 3201 and RHT 3201, standardized to 1 × 10^8^ CFU/mL, was mixed with equal volumes (100 μL) of the pathogenic inoculum. The plates were incubated under pathogen‐specific culture conditions. The optical density at 600 nm was measured using a microplate spectrophotometer, and the anti‐pathogenic activities of *L. rhamnosus* IDCC 3201 and RHT 3201 were quantified based on the inhibition of bacterial growth.

**TABLE 1:** Pathogens used in this study.

Pathogen	Growth medium	Culture conditions
*Escherichia coli* K12	Luria Bertani	37°C, 24 h, aerobic
*Staphylococcus aureus* ATCC 25923	Tryptic soy broth	37°C, 24 h, aerobic
*Enterococcus faecalis* ATCC 49619	Brain heart infusion	37°C, 24 h, aerobic
*Salmonella Typhimurium* ATCC 13311	Nutrient medium	37°C, 24 h, aerobic

### 2.4 Quantification of SCFAs

Quantification of SCFAs was performed as previously described by Kim et al. (2025), with minor modifications. The supernatants from *L. rhamnosus* IDCC 3201 cultures and RHT 3201 were centrifuged at 6000 rpm and 4°C. The samples were filtered with a 0.45‐μm syringe filter. SCFAs (acetic acid, propionic acid, butyric acid, isovaleric acid, and isobutyric acid) and their precursors (succinic acid and lactic acid) were analyzed using high‐performance liquid chromatography (Agilent 1260, Agilent Technologies, CA, USA). For separation and quantification of SCFAs and their precursors, an Aminex HPX‐87H column (9 μm, 300 × 7.8 mm, Bio‐Rad Laboratories, CA, USA) was used. The mobile phase comprised 0.005 N sulfuric acid with a constant flow rate of 0.6 mL/min. The column temperature was maintained at 60°C, and each sample was run for 45 min. Peaks were detected using an ultraviolet detector set at 210 nm, with the detector maintained at 50°C. Finally, SCFAs were quantified based on calibration curves prepared for each SCFA.

### 2.5 Cell Culture Conditions and Sample Preparation

Murine RAW 264.7 macrophages were purchased from the Korean Cell Line Bank (Seoul, Korea) and grown in Dulbecco's modified Eagle's medium (Welgene, Gyeongsan, Korea) supplemented with 10% fetal bovine serum, 100 U/mL penicillin, and 100 μg/mL streptomycin (HyClone, Logan, UT, USA) at 37°C in a 5% CO_2_‐humidified incubator. RHT 3201 was homogenized by for 20 min using a sonicator (Branson Ultrasonics, Danbury, CT, USA). After centrifugation for 10 min, the supernatants were filtered.

### 2.6 Cell Viability Assay

RAW 264.7 cell viability was assessed to determine the cytotoxicity of RHT 3201 using 3‐(4,5‐dimethylthiazol‐2‐yl)‐2,5‐diphenyltetrazolium bromide (MTT). RAW 264.7 cells (1.5 × 10^5^ cells/mL) were seeded in a 96‐well plate and incubated at 37°C for 24 h. The cells were treated with appropriate concentrations of RHT 3201 (0, 0.625, 1.25, 2.5, 5, or 10 × 10^7^ CFU/mL) and stimulated with or without lipopolysaccharide (LPS; 1 μg/mL) for 24 h. After incubation, the culture medium was discarded, and the MTT solution (0.5 mg/mL) was added to each well, followed by incubation at 37°C for 2 h. The formazan crystals were dissolved in dimethyl sulfoxide (DMSO), and the optical density was measured at 590 nm using a microplate reader (BioTek, Winooski, VT, USA).

### 2.7 Real‐Time Quantitative Reverse Transcription Polymerase Chain Reaction (qRT‐PCR)

RAW 264.7 cells were treated with different concentrations of RHT 3201 (0, 2.5, 5, or 10 × 10^7^ CFU/mL) and stimulated with or without LPS (1 μg/mL) for 3 h. Total RNA was extracted using TRIzol reagent (Thermo Fisher Scientific, Waltham, MA, USA) according to the manufacturer's instructions. Total RNA (1 μg) was transcribed to complementary DNA(cDNA) using random hexamers and reverse transcriptase (Superscript IV First‐Strand Synthesis System; Thermo Fisher Scientific) in a total volume of 20 μL. To amplify cDNA, qRT‐PCR was performed with SYBR Green Real‐Time PCR master mix (Toyobo, Osaka, Japan) using the QuantStudio 1 real‐time PCR system (Applied Biosystems, Thermo Fisher Scientific). The amplification protocol included an initial denaturation at 95°C for 10 s, followed by 40 cycles of 95°C for 5 s and 60°C for 30 s. The PCR primer sequences are listed in Table 2. The relative mRNA expression of specific genes was normalized to that of glyceraldehyde‐3‐phosphate dehydrogenase (*Gapdh*) using the 2^−ΔΔ*Ct*^ method.

**TABLE 2:** Primers used in this study.

Gene	Orientation	
*IL‐1β*	Forward	ACCTGGGCTGTCCTGATGAGAG
	Reverse	TGTTGATGTGCTGCTGCGAGAT
*IL‐6*	Forward	GTTCTCTGGGAAATCGTGGA
	Reverse	GGAAATTGGGGTAGGAAGGA
*COX‐2*	Forward	CTGGTGCCTGGTCTGATGATGTATG
	Reverse	TCTCCTATGAGTATGAGTCTGCTGGTT
*TNF‐α*	Forward	AACTAGTGGTGCCAGCCGAT
	Reverse	CTTCACAGAGCAATGACTCC
*GAPDH*	Forward	ACTCCACTCACGGCAAATTC
	Reverse	GTCATGAGCCCTTCCACAAT

Abbreviations: COX‐2, cyclooxygenase‐2; GAPDH, glyceraldehyde‐3‐phosphate dehydrogenase; IL‐1β, interleukin‐1β; IL‐6, interleukin 6; TNF‐α, tumor necrosis factor α.

### 2.8 In Vitro Colonic Fermentation

In vitro colonic fermentation was performed using fecal samples as inocula for bacterial cultures (Daud et al. 2019). Fecal samples were diluted 1:10 (w/v) in phosphate‐buffered saline (PBS; pH 7.4) and homogenized for 10 min using Bag Mixer 400 SW (Interscience, France). A fermentation basal medium was formulated with the following components: 0.01 g/L calcium chloride hexahydrate (CaCl_2_·6H_2_O), 0.04 g/L potassium dihydrogen phosphate (KH_2_PO_4_), 0.01 g/L magnesium sulfate heptahydrate (MgSO_4_·7H_2_O), 2 g/L sodium bicarbonate (NaHCO_3_), 0.1 g/L sodium chloride (NaCl), 0.5 g/L L‐cysteine hydrochloride, 2 mL/L Tween 80, 2 g/L yeast extract, 0.05 g/L hemin, 10 μL/L vitamin K, 0.5 g/L bile salts, and 2 g/L peptone water. The pH was adjusted to 7.0, and resazurin solution (0.025% (w/v)) was added at 4 mL/L. The basal medium was sterilized by autoclaving (Lee et al. 2025).

To prepare the colon medium, 5 mL of fecal slurry was combined with 45 mL of autoclaved basal medium. Subsequently, *L. rhamnosus* IDCC 3201 or RHT 3201 was added to the colon medium at a final concentration of 1.0 × 10^9^ CFU/mL. The fermentation mixture was incubated at 37°C for 24 h. Following incubation, the culture was centrifuged at 8000 rpm for 10 min to separate the bacterial cells from the supernatant. The bacterial pellet was harvested for metagenomic analysis, and the supernatant was analyzed for metabolites.

### 2.9 Metabolome Analysis

#### 2.9.1 Metabolites Extraction

Metabolites were extracted from the supernatants of colonic fermentation samples for metabolomic analysis (Kim et al. 2025). To each 750 μL of sample, 2.25 mL of ice‐cold methanol was added, followed by vortexing for 1 min on ice. The mixture was centrifuged at 10,000 rpm for 10 min at 4°C. The resulting supernatant was collected and filtered through a 0.2‐μm polyvinylidene fluoride syringe filter. A 100 μL aliquot of the filtered supernatant was transferred to a 1.5‐mL microtube for drying. The filtered samples were then dried using a vacuum concentrator (Eppendorf Concentrator Plus, Eppendorf, Hamburg, Germany).

#### 2.9.2 Gas Chromatography (GC)–Mass Spectrometry (MS) Analysis

The dried samples were derivatized by adding 50 μL of a 20 mg/mL methoxyamine hydrochloride solution prepared in pyridine (Sigma, St. Louis, MO, USA) and incubating at 30°C for 90 min (Kim et al. 2025). Subsequently, 100 μL of *N*,*O*‐bis(trimethylsilyl)trifluoroacetamide (Sigma) was added to the samples and heated at 60°C for 30 min. A mixture of alkane standards and fluoranthene was used as the retention index and internal standard, respectively.

GC–MS was performed using a Chromatec‐Crystal GC system (Chromatec, Mari El, Russia) coupled to a Chromatec‐Crystal mass spectrometer (Chromatec). Separation was achieved on a J&W GC column (VF‐5 ms, 60‐m length, 0.25 mm internal diameter, and 0.25‐μm film thickness) (Agilent, Santa Clara, CA, USA). Derivatized samples were injected at 280°C with a split ratio of 1:10, and metabolites were separated using a helium flow rate of 5 mL/min. The oven temperature program was as follows: an initial hold at 50°C for 2 min, followed by an increase to 180°C at 5°C/min; the temperature was held at 180°C for 8 min, increased to 210°C at 2.5°C/min, ramped to 320°C at 5°C/min, and held at 320°C for 10 min.

Mass spectra were collected across a scan range of 35–650 *m*/*z* at an acquisition rate of 0.2 spectra per second in standard mode, with the ion source temperature maintained at 280°C. Data analysis was carried out using Thermo Xcalibur with automated peak detection enabled. Metabolite identification was conducted by comparing mass spectra and retention indices to the NIST Mass Spectral Library (version 2.4, Gaithersburg, MD, USA) and utilizing the MS‐DIAL platform (http://prime.psc.riken.jp/compms/msdial/main.html). Relative metabolite intensities were normalized to the sum of identified peaks.

### 2.10 Metagenome Analysis

#### 2.10.1 16S Full‐Length Metagenomic Library Construction and Sequencing

Total microbial DNA was extracted from each sample using the DNeasy PowerSoil Pro Kit (Qiagen, Hilden, Germany), according to the manufacturer's instructions. DNA quality and integrity were evaluated using the Agilent 2100 Bioanalyzer (Agilent Technologies). Full‐length bacterial 16S rRNA genes were amplified with universal primers 27F (5′‐AGRGTTYGATYMTGGCTCAG‐3′) and 1492R (5′‐RGYTACCTTGTTACGACTT‐3′), both tagged with PacBio barcodes to facilitate multiplexing. The PCR products were quantified and size‐verified via the Agilent 4200 TapeStation system (Waldbronn, Germany), normalized, and pooled in equimolar concentrations. Library preparation was performed using the SMRTbell Express Template Prep Kit 3.0 (Pacific Biosciences, Menlo Park, CA, USA), followed by sequencing on the PacBio Sequel II platform using SMRT Cell 8 M and 10‐h movie captures, according to the manufacturer's full‐length 16S amplicon sequencing protocol. Raw reads were processed into high‐fidelity circular consensus sequences (HiFi reads) using SMRT Link.

#### 2.10.2 16S Metagenomic Data Analysis

Raw CCS reads were imported into QIIME2 and converted to QIIME2 artifact format (.qza). Amplicon sequence variants (ASVs) were determined using the DADA2 plugin, which denoises reads and removes chimeras. Taxonomic classification was performed using the SILVA rRNA database (v.138.99), optimized for long‐read 16S sequences. Relative abundance profiles at the phylum, genus, and species levels were computed for comparative analyses of microbial communities between groups.

Alpha diversity indices were calculated from rarefied ASV feature tables within QIIME2. Between‐group differences in alpha diversity were assessed in R using the Wilcoxon rank‐sum test, and plots were generated with the ggpubr package. Beta diversity was measured using Bray–Curtis distances, and community composition patterns were visualized through nonmetric multidimensional scaling (NMDS). Statistical significance of between‐group differences in community composition was assessed using permutational multivariate analysis of variance (PERMANOVA).

### 2.11 Statistical Analysis

Data are expressed as mean ± standard deviation (SD). Unless otherwise stated, anti‐pathogenic activity, SCFA quantification, and cell‐based assay data were analyzed using an unpaired two‐tailed *t*‐test with GraphPad Prism 10.2.0 (GraphPad Software Inc.). Alpha‐diversity data were analyzed using the Wilcoxon rank‐sum test, and beta‐diversity differences were assessed using PERMANOVA, as described above. Differences were considered significant at *p* < 0.05. Heatmaps of metabolome data were generated using MetaboAnalyst 5.0 (https://www.metaboanalyst.ca/).

## 3 Results

### 3.1 Anti‐Pathogenic Effects of Supernatants Derived From *L. rhamnosus* IDCC 3201 and RHT 3201

**FIGURE 1:**
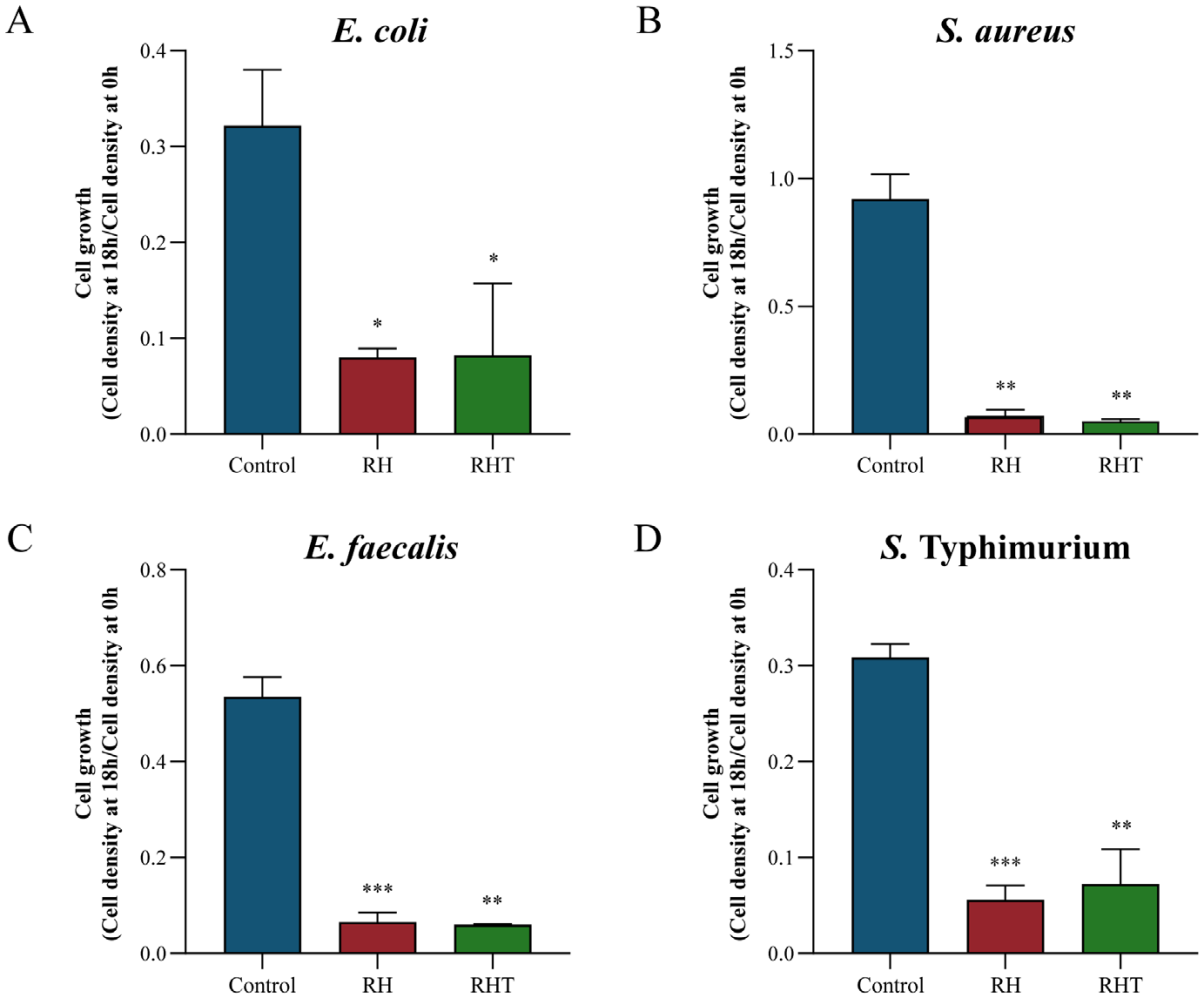
Anti‐pathogenic activities of *L. rhamnosus* IDCC 3201 and heat‐inactivated *L. rhamnosus* IDCC 3201 (RHT 3201) against *Escherichia coli* K12, *Staphylococcus aureus* ATCC 25923, *Enterococcus faecalis* ATCC 49619, and *Salmonella* Typhimurium ATCC 13311. Four pathogenic strains were treated with the cell‐free supernatants of *L. rhamnosus* IDCC 3201 and heat‐inactivated *L. rhamnosus* IDCC 3201 for 24 h. Data are shown as mean ± standard deviation (*n* = 3). Means are significantly different with *p < 0.05, **p < 0.01, and ***p < 0.001 using a *t*‐test compared with the control groups.

To assess whether heat inactivation influences anti‐pathogenic activity, viable *L. rhamnosus* IDCC 3201 and heat‐inactivated RHT 3201 were evaluated. Supernatants obtained at an initial cell density of 1 × 10^8^ CFU/mL from *L. rhamnosus* IDCC 3201 and RHT 3201 effectively inhibited the growth of multiple pathogens (Figure 1). Comparable inhibitory effects were observed between *L. rhamnosus* IDCC 3201 and RHT 3201 against *Escherichia coli* K12 and *Staphylococcus aureus* ATCC 25923. Notably, *L. rhamnosus* IDCC 3201 demonstrated significantly stronger inhibitory effects against *Enterococcus faecalis* ATCC 49619 and *Salmonella* Typhimurium ATCC 13311 than RHT 3201.

### 3.2 SCFA Production by *L. rhamnosus* IDCC 3201 and RHT 3201

The lactic acid concentrations of *L. rhamnosus* IDCC 3201 and RHT 3201 were quantitatively assessed. *L. rhamnosus* IDCC 3201 produced 43.53 mg/g, whereas RHT 3201 demonstrated a markedly higher concentration at 141.18 mg/g (Table 3). This elevated level of lactic acid in the RHT sample is attributable to the vacuum concentration step applied during its manufacturing process, which led to a greater detectable lactic acid content compared to the live bacterial form (*p* < 0.001).

**TABLE 3:** Production of lactic acid by each supernatant of *Lacticaseibacillus rhamnosus* IDCC 3201 and RHT 3201.

	Lactic acid (mg/g)	*p*
RH 3201	43.53 ± 2.18	< 0.001
RHT 3201	141.18 ± 3.65	

### 3.3 Cytotoxicity of RHT 3201 Toward LPS‐Induced RAW 264.7 Cells

**FIGURE 2:**
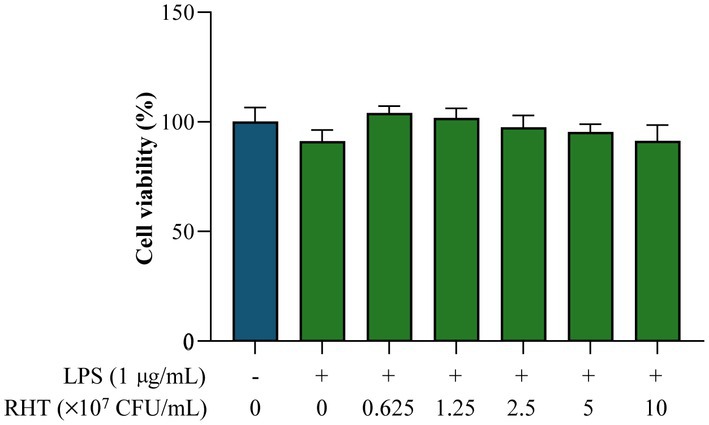
Viability of RAW264.7 cells. Cells were treated with different concentrations of RHT 3201 (0, 2.5, 5, or 10 × 10^7^ CFU/mL) and stimulated with LPS (1 μg/mL) for 24 h. Data are expressed as the mean ± standard deviation. Statistical significance between groups was determined using an unpaired two‐tailed *t*‐test.

The cytotoxic effects of RHT 3201 were investigated using an MTT assay. No significant reduction in the viability of RAW 264.7 cells treated with LPS only (1 μg/mL) compared with untreated controls (Figure 2). Additionally, there were no significant differences in the viability of LPS‐induced RAW 264.7 cells treated with various concentrations of RHT 3201 (0, 0.625, 1.25, 2.5, 5, or 10 × 10^7^ CFU/mL) compared with the untreated control. Given that RHT 3201 concentration did not affect the viability of RAW 264.7 cells treated with LPS (1 μg/mL), the three highest concentrations (2.5, 5, or 10 × 10^7^ CFU/mL) were selected for subsequent analyses.

### 3.4 Inflammatory Mediator mRNA Expression in LPS‐Induced RAW 264.7 Cells Treated With RHT 3201

**FIGURE 3:**
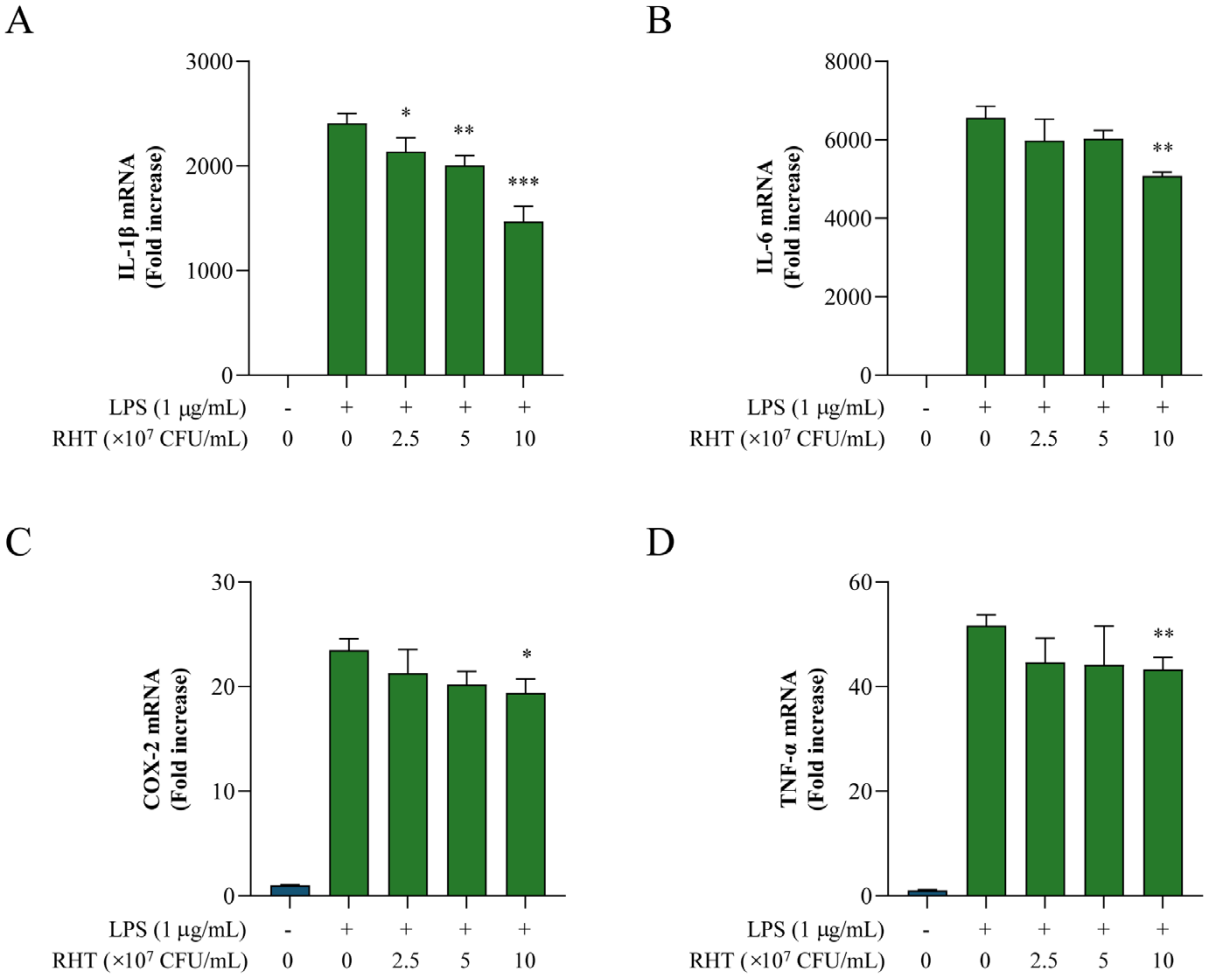
RHT 3201 suppresses LPS‐induced pro‐inflammatory cytokine and mediator expression in RAW 264.7 cells. RAW 264.7 macrophages were pretreated with RHT 3201 at varying concentrations (0, 2.5, 5, or 10 × 10^7^ CFU/mL) and subsequently stimulated with LPS (1 μg/mL) for 3 h. mRNA expression levels of (A) IL‐1β, (B) IL‐6, (C) COX‐2, and (D) TNF‐α were quantified by qRT‐PCR. Data are presented as mean ± standard deviation (SD) of at least three independent experiments. Statistical significance compared with the LPS‐only control group was assessed using an unpaired two‐tailed *t*‐test (*p < 0.05, **p < 0.01, ***p < 0.001).

A previous study reported anti‐inflammatory effects of *L. rhamnosus* IDCC 3201 supernatants in LPS‐stimulated RAW 264.7 cells (Chae et al. 2022). In the present study, transcript‐level anti‐inflammatory activity was evaluated only for RHT 3201. Therefore, the mRNA levels of inflammatory mediators (IL‐1β, IL‐6, COX‐2, and TNF‐α) were measured in LPS‐induced RAW 264.7 cells treated with or without RHT 3201 (2.5, 5, or 10 × 10^7^ CFU/mL) using qRT‐PCR. Compared with the LPS‐only group, RHT 3201 reduced the mRNA expression of IL‐1β, IL‐6, COX‐2, and TNF‐α (Figure 3). At 10 × 10^7^ CFU/mL, expression of these mediators decreased by approximately 39%, 23%, 17%, and 16%, respectively. IL‐1β showed the clearest dose‐dependent reduction (Figure 3A), whereas reductions in IL‐6, COX‐2, and TNF‐α at 2.5 and 5 × 10^7^ CFU/mL did not reach statistical significance (Figure 3B–D).

### 3.5 Distinct Metabolic Profiles Induced by *L. rhamnosus* IDCC 3201 and RHT 3201 in IBD Fecal Sample Fermentation

To investigate the metabolic effects of *L. rhamnosus* IDCC 3201 and RHT 3201, we analyzed the metabolite profiles of three experimental groups: (1) control (fermented IBD fecal samples alone), (2) *L. rhamnosus* IDCC 3201 (fermented IBD fecal samples with viable *L. rhamnosus* IDCC 3201), and (3) RHT 3201 (fermented IBD fecal samples with heat‐inactivated RHT 3201).

**FIGURE 4:**
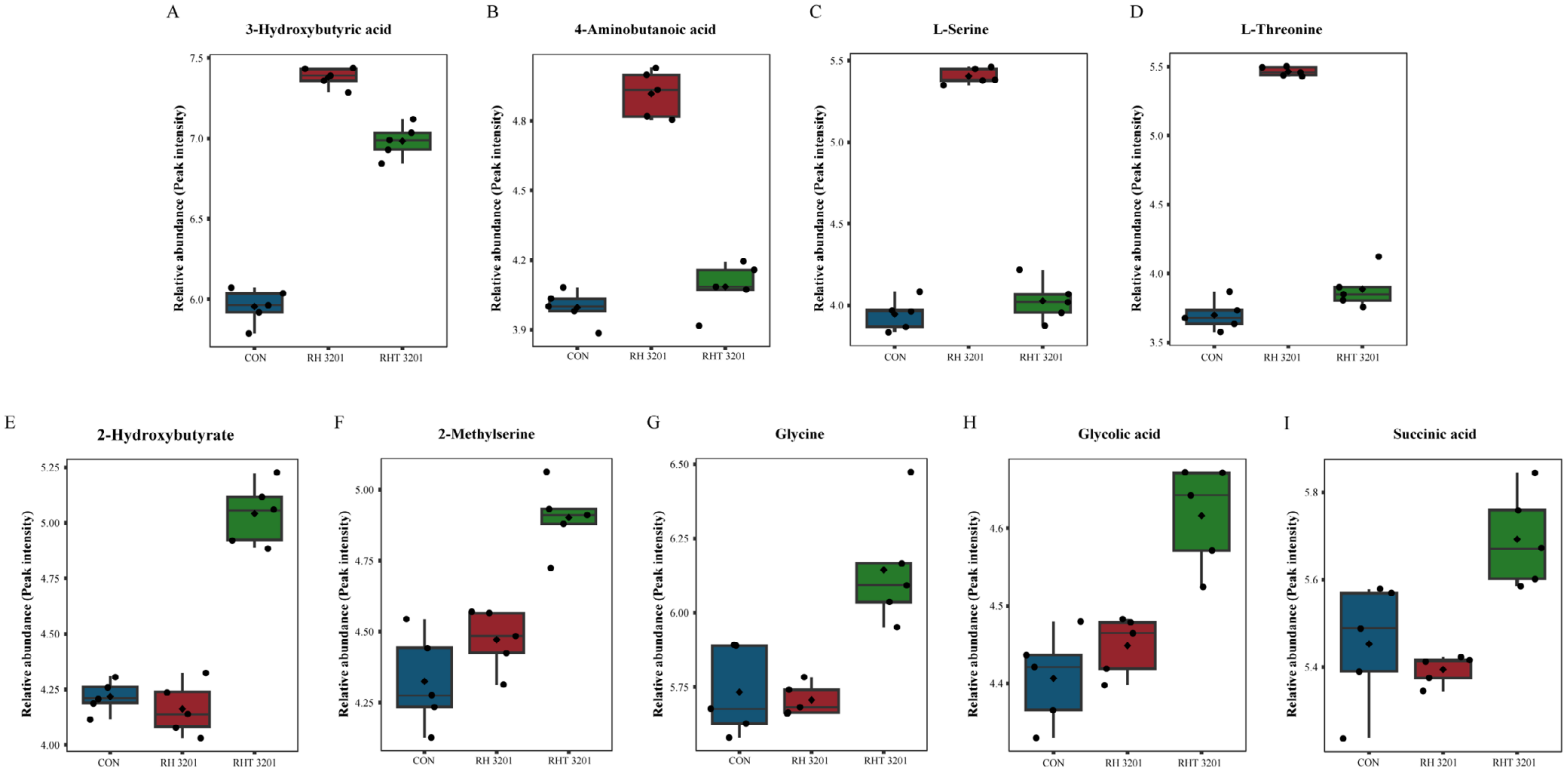
Differential metabolite abundances in fecal samples treated with *Lacticaseibacillus rhamnosus* IDCC 3201 (RH 3201) and its heat‐inactivated form (RHT 3201). (A–D) Metabolites were significantly enriched in the RH 3201 group compared with the control (CON) and RHT 3201, including 3‐hydroxybutyric acid (A), 4‐aminobutanoic acid (B), l‐serine (C), and l‐threonine (D). (E–I) Metabolites significantly enriched in the RHT 3201 group compared with CON and RH 3201, including 2‐hydroxybutyrate (E), 2‐methylserine (F), glycine (G), glycolic acid (H), and succinic acid (I). Boxplots represent median (line), mean (diamond), interquartile range (box), and minimum/maximum values (whiskers); individual data points are shown as dots.

Distinct differences were detected in the metabolite profiles of the groups. In the *L. rhamnosus* IDCC 3201 group, significant increases in 3‐hydroxybutyric acid, 4‐aminobutanoic acid (GABA), l‐serine, and l‐threonine levels were observed. Conversely, the RHT 3201 group showed a distinct increase in 2‐hydroxybutyrate, 2‐methylserine, glycine, glycolic acid, and succinic acid levels (Figure 4).

**FIGURE 5:**
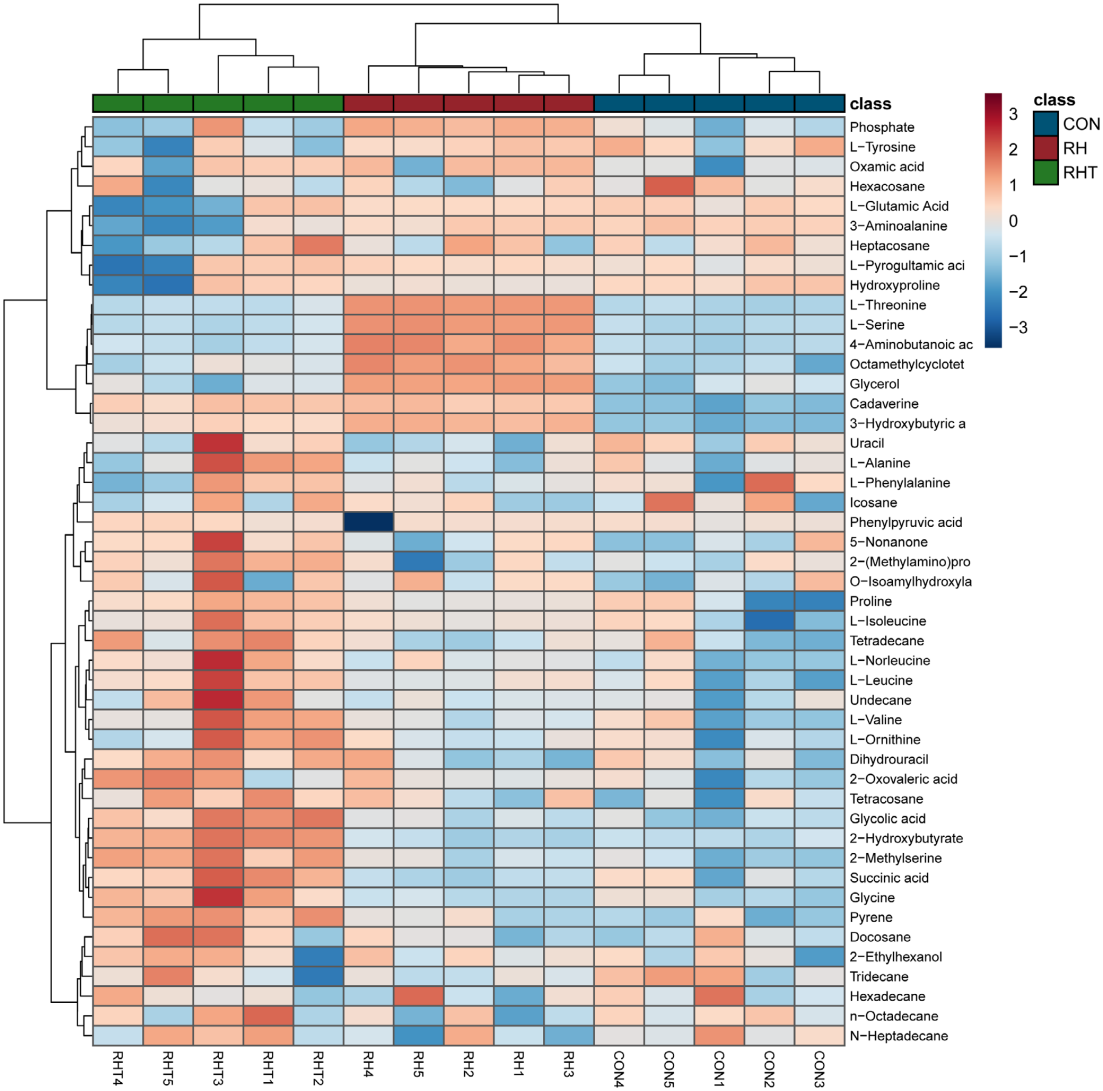
Relative abundance of metabolites across different experimental groups. RHT 3201 (green), RH 3201 (red), and control (CON, blue). Rows correspond to individual metabolites, and columns represent biological replicates for each group. Metabolite levels are standardized (*z*‐score), with red indicating higher relative abundance and blue indicating lower relative abundance. Hierarchical clustering was applied both to metabolites (rows) and samples (columns), highlighting distinct metabolic profiles among the three groups. Key amino acids, organic acids, and lipid‐related metabolites are shown, illustrating differential metabolic responses induced by viable and heat‐inactivated *Lacticaseibacillus rhamnosus* IDCC 3201 treatments.

These differences were further highlighted in a heatmap analysis, which visually confirmed that *L. rhamnosus* IDCC 3201 and RHT 3201 exhibited distinct metabolic shifts compared with the control group and with each other (Figure 5). These results reflected the specific metabolic effects induced by *L. rhamnosus* IDCC 3201 or RHT 3201.

### 3.6 Fecal Microbiota Profile After Treatment With *L. rhamnosus* IDCC 3201 or RHT 3201

**FIGURE 6:**
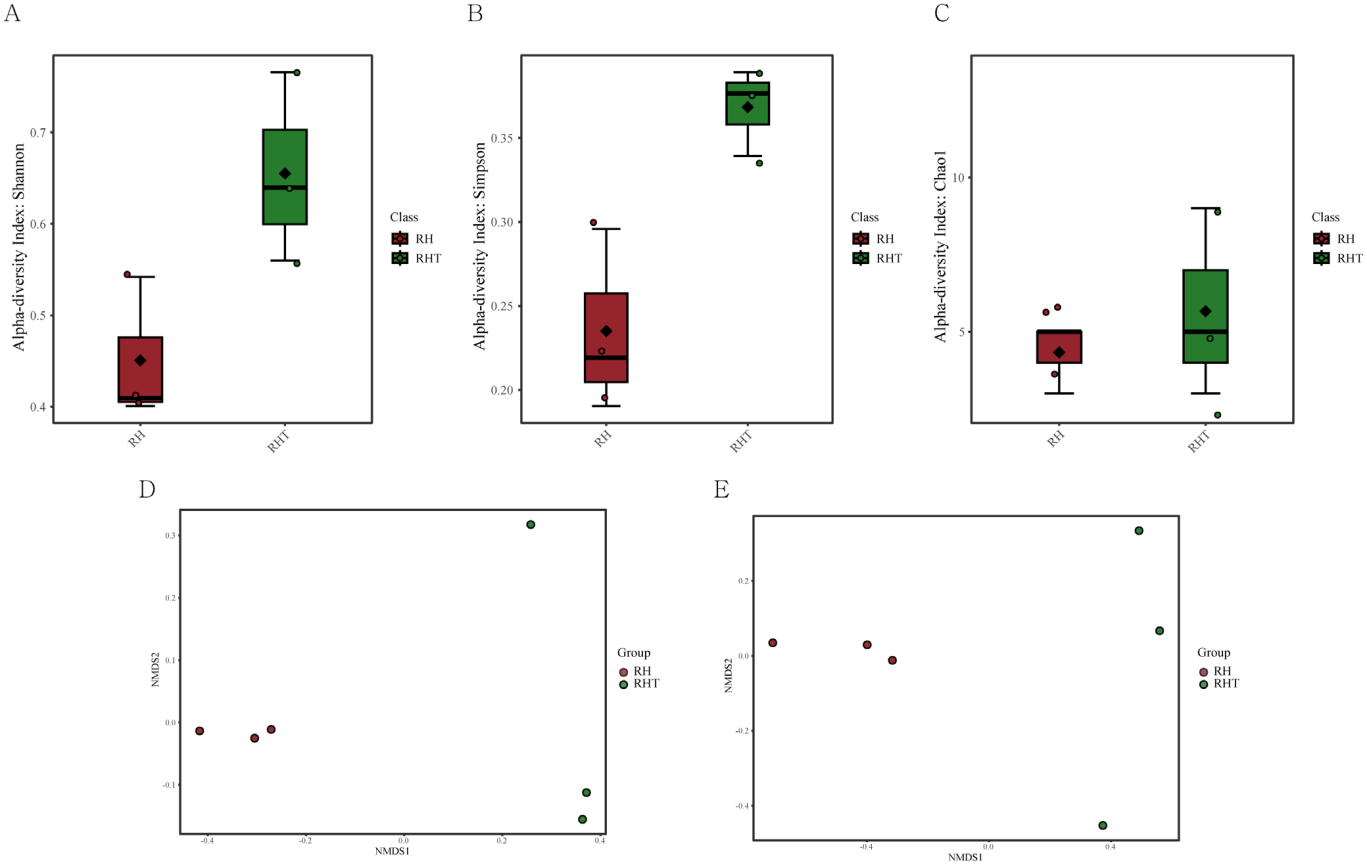
Alpha and beta diversity of the fecal microbiota in *Lacticaseibacillus rhamnosus* IDCC 3201 (RH) and heat‐inactivated RHT 3201 (RHT). Alpha‐diversity was assessed using the (A) Shannon, (B) Simpson, and (C) Chao1 indices. Beta‐diversity was visualized using nonmetric multidimensional scaling (NMDS) ordination based on species‐level fecal microbiota profiles, shown in (D) and (E). Each point represents an individual sample.

To evaluate changes in the overall microbial community, alpha‐ and beta‐diversity were compared between the *L. rhamnosus* IDCC 3201 and RHT 3201 groups (Figure 6). The RHT group exhibited higher alpha diversity than the IDCC 3201 group, as indicated by Shannon and Simpson indices. Chao 1 richness was also generally greater for the RHT group, along with greater inter‐individual variability. Additionally, NMDS ordination revealed a separation trend between the IDCC 3201 and RHT groups, suggesting differences in overall microbial community structure.

**FIGURE 7:**
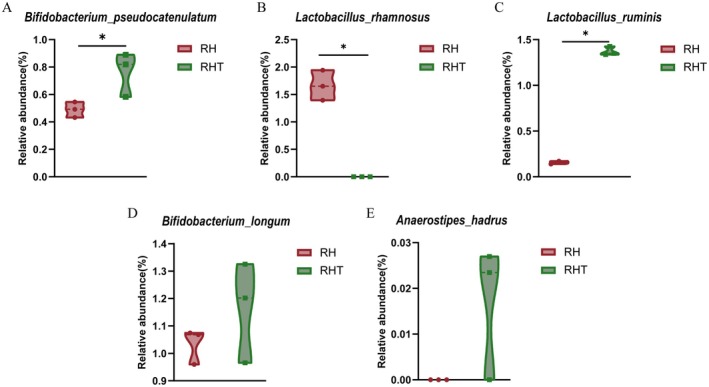
Comparison of fecal microbiota composition at the species level between *Lacticaseibacillus rhamnosus* IDCC 3201 (RH) and heat‐inactivated RHT 3201 (RHT) groups. Relative abundances of key bacterial species are shown, including (A) *Bifidobacterium pseudocatenulatum* , (B) *Lactobacillus rhamnosus* , (C) *Ligilactobacillus ruminis*, (D) *Bifidobacterium longum* , and (E) *Anaerostipes hadrus*. Data are expressed as mean relative abundance (%). Statistical significance is indicated where applicable (*p < 0.05; exact *p* values shown for nonsignificant comparisons). Violin plots illustrate the distribution and variability of the data within each group.

The fecal microbiota composition was analyzed at the species level in the *L. rhamnosus* IDCC 3201 and RHT 3201 groups (Figure 7). The relative abundance of *B. pseudocatenulatum* was significantly higher in the RHT 3201 group than in the IDCC 3201 group (*p* < 0.05). *Ligilactobacillus ruminis* was also significantly enriched in the RHT 3201 group compared with the IDCC 3201 group (*p* < 0.05). In contrast, *L. rhamnosus* was detected only in the IDCC 3201 group (*p* < 0.05) during the 24‐h ex vivo fermentation period. Although the relative abundance of *Bifidobacterium longum* tended to be higher in the RHT 3201 group, this difference was not statistically significant (*p* = 0.275). Similarly, *Anaerostipes hadrus* was more abundant in the RHT 3201 group, but this difference did not reach statistical significance (*p* = 0.121).

## 4 Discussion

This study investigated the anti‐pathogenic activities of viable and heat‐inactivated *L. rhamnosus* IDCC 3201 against *E. coli* K12, *S. aureus* ATCC 25923, *E. faecalis* ATCC 49619, and *S. Typhimurium* ATCC 13311. Both *L. rhamnosus* IDCC 3201 and RHT 3201 exhibited similar inhibitory effects against *E. coli* K12 and *S. aureus* ATCC 25923, suggesting that some inhibitory components remain active after heat inactivation (Piqué et al. 2019). In contrast, *E. faecalis* ATCC 29212 and *S. typhimurium* ATCC 13311 were more strongly inhibited by *L. rhamnosus* IDCC 3201 than by RHT 3201. This difference may reflect viability‐dependent metabolic activity or other labile factors that were reduced during heat inactivation. Accordingly, the two preparations appeared to show different inhibitory profiles depending on the target pathogen.

These results suggest that the relative performance of viable and heat‐inactivated preparations may vary according to the pathogen evaluated (Fijan et al. 2022). Within the limits of the current in vitro assay, RHT 3201 showed comparable activity against *E. coli* K12 and *S. aureus* ATCC 25923, whereas viable *L. rhamnosus* IDCC 3201 showed strong inhibition against *E. faecalis* ATCC 29212 and *S. typhimurium* ATCC 13311.

The SCFA results are consistent with the presence of lactic acid observed in *L. rhamnosus* GG and *L. rhamnosus* SD11 (Jang et al. 2021; Thananimit et al. 2022). The SCFA‐related findings should be interpreted cautiously. In the present study, the most prominent difference between preparations was the higher lactic acid content measured in RHT 3201. As described above, this difference likely reflects the vacuum concentration and downstream processing steps used during preparation rather than de novo metabolic activity after heat inactivation. Similar accumulation of product‐associated metabolites has been reported in processed heat‐inactivated formulations and may reflect technological features of manufacture rather than direct physiological activity (Lee et al. 2025). Therefore, lactic acid measured in the product should be interpreted as a preformed, product‐associated component and not as a surrogate for colonic lactic acid production after ingestion.

Because fecal or intestinal organic acid levels were not measured in vivo, the present data do not support direct conclusions regarding host SCFA biology or in vivo lactic acid exposure following administration of RHT 3201 versus live bacteria.

Macrophages activated by LPS produce pro‐inflammatory cytokines, such as IL‐1 and IL‐6 (Lee and Choi 2018). However, excessive production of these cytokines can lead to tissue damage and inflammatory diseases (Kany et al. 2019). In LPS‐stimulated RAW 264.7 cells, RHT 3201 reduced the mRNA expression of IL‐1β, IL‐6, COX‐2, and TNF‐α. These findings are consistent with prior in vitro reports involving *L. rhamnosus* ‐derived preparations (Chae et al. 2022), but they should be interpreted within the limits of the current assay. In the present study, anti‐inflammatory activity was evaluated only for RHT 3201 and only at the transcript level; therefore, the data do not establish a direct head‐to‐head comparison with live IDCC 3201 or confirm downstream protein or cytokine secretion responses. Additional experiments, such as matched live‐versus‐heat‐inactivated assays, ELISA, and Western blotting, will be required to determine whether these transcript‐level changes translate into functional anti‐inflammatory effects.

The metabolic effects of *L. rhamnosus* IDCC 3201 and RHT 3201 were examined in a single‐donor IBD fecal fermentation model. Significant increases in 3‐hydroxybutyric acid, GABA, l‐serine, and l‐threonine levels were observed with *L. rhamnosus* IDCC 3201. These metabolites reflect differences in amino acid metabolism and microbial fermentation, but the present data do not establish a direct mechanistic link to host outcomes (Bai et al. 2024; Deng et al. 2024; Mao et al. 2011).

In contrast, RHT 3201 was associated with higher levels of 2‐hydroxybutyrate, 2‐methylserine, glycine, glycolic acid, and succinic acid, indicating a distinct metabolite profile. These differences may reflect altered substrate availability, processing‐derived components, or shifts in microbial activity in response to the heat‐inactivated preparation rather than a defined host‐directed mechanism (Tajima et al. 2015; Wang et al. 2013). These findings indicate that IDCC 3201 and RHT 3201 were associated with distinct metabolite patterns in the donor‐derived ex vivo fermentation system; whether these differences translate to host effects requires further validation.

Metagenomic analysis also suggested distinct effects of *L. rhamnosus* IDCC 3201 and RHT 3201 on fecal microbiota composition in the ex vivo fermentation model. RHT 3201 showed higher alpha‐diversity indices (Shannon/Simpson) and a separation trend from IDCC 3201 in NMDS ordination, suggesting differences in overall community structure. Together with the species‐level observations, these data support distinct microbiota‐associated profiles of viable and heat‐inactivated preparations in this single‐donor ex vivo setting.

RHT 3201 was associated with a higher relative abundance of *B. pseudocatenulatum* and *L. ruminis* and with non‐significant upward trends in *B. longum* and *A. hadrus*. These observations are consistent with prior reports that heat‐inactivated probiotic preparations can alter ex vivo or in vitro microbial community structure (Vinderola et al. 2022). In contrast, *L. rhamnosus* was detected only in the IDCC 3201 group during the 24‐h fermentation period, indicating short‐term detectability under the ex vivo culture conditions. These findings should not be interpreted as evidence of host colonization, long‐term persistence, or in vivo engraftment, which cannot be determined from the present fecal fermentation model. Together, the metabolite and community analyses indicate distinct ex vivo profiles for the viable and heat‐inactivated preparations, but they should not be interpreted as direct evidence of efficacy in a host.

A key limitation of this study is that the in vitro fecal fermentation experiment was conducted using stool from a single adult donor with IBD. Therefore, the observed metabolomic and microbiota changes, including diversity indices and species‐level shifts, should be interpreted as exploratory and donor‐specific findings within this ex vivo model. Although repeated fermentations were performed, these represent experimental replicates and do not substitute for independent biological replication across donors. Future study including multiple independent donors will be necessary to validate the reproducibility and biological generalizability of the observed responses. Importantly, the metabolomic findings in the present fecal fermentation model should be interpreted as correlative rather than mechanistic, because no epithelial barrier assays, host‐cell rescue experiments, or protein‐level immune readouts were performed to directly link the observed metabolite shifts to host benefit. Moreover, an important limitation of the present comparison is that RHT 3201 was not generated as a viability‐matched control differing from live IDCC 3201 only by heat inactivation. Rather, it represents a heat‐inactivated formulation that also underwent downstream processing steps such as concentration, excipient blending, freeze‐drying, and milling. Therefore, the observed differences between IDCC 3201 and RHT 3201 may reflect the combined effects of viability loss, preformed metabolites, and manufacturing and formulation processes and should not be interpreted as being driven solely by viability status.

## 5 Conclusion

In summary, viable *L. rhamnosus* IDCC 3201 and its heat‐inactivated formulation, RHT 3201, showed comparable anti‐pathogenic activity against some tested pathogens but a distinct ex vivo microbiota‐ and metabolite‐associated profile. RHT 3201 also showed transcript‐level immunomodulatory activity in LPS‐stimulated RAW 264.7 cells. Because the fermentation experiment used a single donor, the inflammatory assessment was limited to mRNA expression, and the comparison may also reflect differences introduced by downstream processing of the heat‐inactivated formulation; these findings should be interpreted cautiously. Additional multi‐donor, protein‐level, and in vivo studies are needed to determine the reproducibility and physiological relevance of these observations.

## Author Contributions

**Hayoung Kim:** conceptualization, investigation, methodology, writing – original draft, visualization. **Hanbin Lee:** methodology, investigation, writing – original draft. **Won Yeong Bang:** methodology, investigation, writing – original draft. **Haeseong Park:** writing – review and editing. **Jin Seok Moon:** conceptualization, project administration, writing – review and editing.

## Funding

The authors have nothing to report.

## Ethics Statement

The fecal sample collection protocol was reviewed and approved by the Institutional Review Board of Songdo Hospital (approval number: 2021‐004).

## Conflicts of Interest

The authors declare no conflicts of interest.

## Data Availability

The data that support the findings of this study are available on request from the corresponding author.

## References

[fsn371780-bib-0001] Antunes, C. , K. Dziadkowiec , and A. Charabaty . 2021. “Advances in Our Understanding of the Pathogenesis of Inflammatory Bowel Disease.” In Inflammatory Bowel Disease: Pathogenesis, Diagnosis and Management, edited by R. Rajapakse , 1–23. Springer.

[fsn371780-bib-0002] Bai, J. D. K. , S. Saha , M. C. Wood , et al. 2024. “Serine Supports Epithelial and Immune Cell Function in Colitis.” American Journal of Pathology 194, no. 6: 927–940. 38417696 10.1016/j.ajpath.2024.01.021 PMC12178392

[fsn371780-bib-0003] Bron, P. A. , M. Kleerebezem , R. J. Brummer , et al. 2017. “Can Probiotics Modulate Human Disease by Impacting Intestinal Barrier Function?” British Journal of Nutrition 117, no. 1: 93–107. 28102115 10.1017/S0007114516004037 PMC5297585

[fsn371780-bib-0004] Chae, S. A. , S. R. Ramakrishnan , T. Kim , et al. 2022. “Anti‐Inflammatory and Anti‐Pathogenic Potential of *Lacticaseibacillus rhamnosus* IDCC 3201 Isolated From Feces of Breast‐Fed Infants.” Microbial Pathogenesis 173: 105857. 36397614 10.1016/j.micpath.2022.105857

[fsn371780-bib-0005] Daud, N. A. , S. R. Sarbini , A. S. Babji , S. Mohamad Yusop , and S. J. Lim . 2019. “Characterization of Edible Swiftlet's Nest as a Prebiotic Ingredient Using a Simulated Colon Model.” Annals of Microbiology 69, no. 12: 1235–1246.

[fsn371780-bib-0006] Deng, Z. , D. Li , L. Wang , J. Lan , J. Wang , and Y. Ma . 2024. “Activation of GABABR Attenuates Intestinal Inflammation by Reducing Oxidative Stress Through Modulating the TLR4/MyD88/NLRP3 Pathway and Gut Microbiota Abundance.” Antioxidants 13, no. 9: 1141. 39334800 10.3390/antiox13091141 PMC11428452

[fsn371780-bib-0007] Fijan, S. , P. Kocbek , A. Steyer , P. M. Vodičar , and M. Strauss . 2022. “The Antimicrobial Effect of Various Single‐Strain and Multi‐Strain Probiotics, Dietary Supplements or Other Beneficial Microbes Against Common Clinical Wound Pathogens.” Microorganisms 10, no. 12: 2518. 36557771 10.3390/microorganisms10122518 PMC9781324

[fsn371780-bib-0008] Hardy, H. , J. Harris , E. Lyon , J. Beal , and A. D. Foey . 2013. “Probiotics, Prebiotics and Immunomodulation of Gut Mucosal Defences: Homeostasis and Immunopathology.” Nutrients 5, no. 6: 1869–1912. 23760057 10.3390/nu5061869 PMC3725482

[fsn371780-bib-0009] Hill, C. , F. Guarner , G. Reid , et al. 2014. “Expert Consensus Document: The International Scientific Association for Probiotics and Prebiotics Consensus Statement on the Scope and Appropriate Use or the Term Probiotic.” Nature Reviews Gastroenterology & Hepatology 11, no. 8: 506–514. 24912386 10.1038/nrgastro.2014.66

[fsn371780-bib-0010] Jalali, S. , N. Mojgani , M. R. Sanjabi , S. Saremnezhad , and S. Haghighat . 2024. “Functional Properties and Safety Traits of *L. rhamnosus* and *L. reuteri* Postbiotic Extracts.” AMB Express 14, no. 1: 114. 39384663 10.1186/s13568-024-01768-3 PMC11465093

[fsn371780-bib-0011] Jang, H. J. , N. K. Lee , and H. D. Paik . 2021. “ *Lactobacillus plantarum* G72 Showing Production of Folate and Short‐Chain Fatty Acids.” Microbiology and Biotechnology Letters 49: 18–23.

[fsn371780-bib-0012] Jeong, K. , M. Kim , S. A. Jeon , Y.‐H. Kim , and S. Lee . 2020. “A Randomized Trial of *Lactobacillus rhamnosus* IDCC 3201 Tyndallizate (RHT3201) for Treating Atopic Dermatitis.” Pediatric Allergy and Immunology 31, no. 7: 783–792. 32363613 10.1111/pai.13269

[fsn371780-bib-0013] Kany, S. , J. T. Vollrath , and B. Relja . 2019. “Cytokines in Inflammatory Disease.” International Journal of Molecular Sciences 20, no. 23: 6008. 31795299 10.3390/ijms20236008 PMC6929211

[fsn371780-bib-0014] Kim, H. , H. J. Jeon , H. M. Jeong , et al. 2025. “Modulation of the Gut Microbiome and Metabolomes by Fermentation Using a Probiotic Complex in a Dysbiosis Associated Fecal Model.” Journal of Microbiology and Biotechnology 35: e2506014. 41309379 10.4014/jmb.2506.06014 PMC12685591

[fsn371780-bib-0015] Kwon, H. , E. H. Nam , H. Kim , et al. 2024. “Effect of *Lacticaseibacillus rhamnosus* IDCC 3201 on Irritable Bowel Syndrome With Constipation: A Randomized, Double‐Blind, and Placebo‐Controlled Trial.” Scientific Reports 14, no. 1: 22384. 39333245 10.1038/s41598-024-72887-x PMC11437119

[fsn371780-bib-0016] Lee, C. H. , and E. Y. Choi . 2018. “Macrophages and Inflammation.” Journal of Rheumatic Diseases 25, no. 1: 11.

[fsn371780-bib-0017] Lee, J. W. , S. E. Song , I. B. Kim , D. G. Oh , D. J. Kim , and C. H. Kim . 2025. “Functional Properties of Heat‐Killed Lactic Acid Bacteria Isolated From Vietnamese Feces.” Food Science of Animal Resources 45, no. 3: 890–909. 40657516 10.5851/kosfa.2025.e22 PMC12246904

[fsn371780-bib-0018] Mao, X. , X. Zeng , S. Qiao , G. Wu , and D. Li . 2011. “Specific Roles of Threonine in Intestinal Mucosal Integrity and Barrier Function.” Frontiers in Bioscience 3, no. 4: 1192–1200. 10.2741/e322 21622125

[fsn371780-bib-0019] Pham, V. T. , and M. H. Mohajeri . 2018. “The Application of In Vitro Human Intestinal Models on the Screening and Development of Pre‐and Probiotics.” Beneficial Microbes 9, no. 5: 725–742. 29695182 10.3920/BM2017.0164

[fsn371780-bib-0020] Piqué, N. , M. Berlanga , and D. Miñana‐Galbis . 2019. “Health Benefits of Heat‐Killed (Tyndallized) Probiotics: An Overview.” International Journal of Molecular Sciences 20, no. 10: 2534. 31126033 10.3390/ijms20102534 PMC6566317

[fsn371780-bib-0021] Ramos, G. P. , and K. A. Papadakis . 2019. “Mechanisms of Disease: Inflammatory Bowel Diseases.” Mayo Clinic Proceedings 94, no. 1: 155–165. 30611442 10.1016/j.mayocp.2018.09.013 PMC6386158

[fsn371780-bib-0022] Sanders, M. E. , D. J. Merenstein , G. Reid , G. R. Gibson , and R. A. Rastall . 2019. “Probiotics and Prebiotics in Intestinal Health and Disease: From Biology to the Clinic.” Nature Reviews Gastroenterology & Hepatology 16, no. 10: 605–616. 31296969 10.1038/s41575-019-0173-3

[fsn371780-bib-0023] Tajima, Y. , Y. Yamamoto , K. Fukui , et al. 2015. “Impact of an Energy‐Conserving Strategy on Succinate Production Under Weak Acidic and Anaerobic Conditions in *Enterobacter aerogenes* .” Microbial Cell Factories 14, no. 1: 80. 26063229 10.1186/s12934-015-0269-6 PMC4464251

[fsn371780-bib-0024] Thananimit, S. , N. Pahumunto , and R. Teanpaisan . 2022. “Characterization of Short Chain Fatty Acids Produced by Selected Potential Probiotic *Lactobacillus* Strains.” Biomolecules 12, no. 12: 1829. 36551257 10.3390/biom12121829 PMC9775007

[fsn371780-bib-0025] Vinderola, G. , M. E. Sanders , S. Salminen , and H. Szajewskaw . 2022. “Postbiotics: The Concept and Their Use in Healthy Populations.” Frontiers in Nutrition 9: 1002213. 36570166 10.3389/fnut.2022.1002213 PMC9780264

[fsn371780-bib-0026] Wang, W. , Z. Wu , Z. Dai , Y. Yang , J. Wang , and G. Wu . 2013. “Glycine Metabolism in Animals and Humans: Implications for Nutrition and Health.” Amino Acids 45, no. 3: 463–477. 23615880 10.1007/s00726-013-1493-1

[fsn371780-bib-0027] Zhang, X. F. , X. X. Guan , Y. J. Tang , et al. 2021. “Clinical Effects and Gut Microbiota Changes of Using Probiotics, Prebiotics or Synbiotics in Inflammatory Bowel Disease: A Systematic Review and Meta‐Analysis.” European Journal of Nutrition 60, no. 5: 2855–2875. 33555375 10.1007/s00394-021-02503-5

